# Health Qigong engagement scale and wellbeing in Chinese older adults

**DOI:** 10.3389/fpubh.2026.1889948

**Published:** 2026-09-23

**Authors:** Jingwu Liu, Faxiang Fan

**Affiliations:** 1 Department of Physical Education, Changzhi University, Changzhi, Shanxi, China; 2 College of Physical Education, Qiannan Normal University for Nationalities, Duyun, China

**Keywords:** Health Qigong engagement, older adults, perceived social support, psychological wellbeing, resilience, scale development

## Abstract

**Background:**

China's population is aging. Psychological wellbeing describes positive psychological functioning, including self-acceptance, purpose in life, personal growth, positive relationships, autonomy, and the ability to manage daily life. Health Qigong is a traditional Chinese mind-body exercise that combines movement, breathing, and mental focus. However, no specific tool is available to assess how regularly, attentively, and independently older adults engage in Health Qigong. This study developed such a tool and examined its associations with resilience, perceived social support, and psychological wellbeing.

**Methods:**

Guided by self-determination theory, which explains how autonomy, competence, and social connection support sustained behavior, we developed the Health Qigong Engagement Scale through interviews with older practitioners, expert review, and tests of reliability and validity. We then examined data from 1,173 older Health Qigong practitioners in six Chinese cities. The analysis evaluated the scale and examined the cross-sectional associations among engagement, resilience, perceived social support, and psychological wellbeing.

**Results:**

The 18-item scale included three dimensions: continuous behavioral engagement, normative focused engagement, and autonomous persistent engagement. It showed satisfactory reliability and validity. Greater Health Qigong engagement was associated with higher resilience, perceived social support, and psychological wellbeing. The indirect association through resilience accounted for 27.97% of the total association between engagement and psychological wellbeing. The association between engagement and resilience was stronger at higher levels of perceived social support.

**Conclusion:**

The scale provides a practical tool for assessing Health Qigong engagement among Chinese older adults. Greater engagement was associated with better psychological wellbeing, and resilience and perceived social support may help explain this pattern.

## 1 Introduction

China's population is aging rapidly, and the number of older adults continues to grow (1). Along with degenerative changes in physiological functioning, the marginal transition of social roles, and the restructuring of family support networks, older adults generally face multiple negative psychological effects, including an increased sense of dependence induced by physical decline, intensified loneliness caused by shrinking social participation, and existential anxiety arising from weakened self-efficacy (2, 3). The continued accumulation and difficulty of resolving such negative emotional states directly results in a profound erosion of the individual's inner spiritual core in later life (4). It undermines a stable sense of self-worth, dissolves a sense of direction toward future life, and to a large extent weakens older adults' inner belief in coping with everyday adversity (5). When this loss of inner spiritual resources reaches a certain degree, the limitations of focusing only on the treatment of physical illness or the satisfaction of material conditions become apparent, because the quality of later life depends at a deeper level on individuals' positive evaluation and inner experience of their own state of existence (6–8). For this reason, as a core indicator of positive psychological functioning and inner peace, PWB has significance in aging research that goes beyond mere emotional regulation (9, 10). What PWB concerns is not short-term pleasurable experience, but a deep confirmation rooted in self-acceptance, purpose in life, environmental mastery, and personal growth (11). It reflects the inner harmony and vitality that individuals can still maintain in late life even when facing objective limitations (12). In view of this, focusing academic attention on the influencing factors and promotion mechanisms of PWB among older adults is not only an inevitable requirement for responding to the profound challenges of an aging society, but also a key entry point for exploring the practical pathways of positive aging (13–16).

### 1.1 The relationship between HQE and PWB

Health Qigong is a unique form of mind-body cultivation rooted in the philosophical and medical traditions of Chinese health preservation (17). In the context of Chinese indigenous culture, Health Qigong is defined as a trinity form of exercise, with bodily movement as its external carrier, breathing regulation as its internal rhythm, and mental regulation as its spiritual pivot (18, 19). Its core mechanism lies in achieving the smooth circulation of qi and blood through the meridians and the dynamic balance of yin and yang in the internal organs through the coordinated operation of “regulating the body, regulating the breath, and regulating the mind (20–22).” Compared with the Western sport paradigm, which places greater emphasis on external physical fitness indicators such as muscular strength, speed, and endurance, the internal value orientation of Health Qigong is more inclined toward a process of mind-body cultivation characterized by relaxation, tranquility, naturalness, and the unity of body and mind (23–25). Its movements are often marked by slowness, continuity, roundness, and smooth integration (26). In recent years, government-led national fitness initiatives have encouraged regular physical activity across the population. Together with renewed interest in traditional health practices, these initiatives have made Health Qigong increasingly popular in community settings, especially among older adults (27–30). Practicing traditional routines such as Baduanjin and Wuqinxi has already become an important part of the daily leisure life of many older adults (31). Existing studies generally confirm that regular Health Qigong practice has positive effects on physical health dimensions among older adults, such as balance control, flexibility, and cardiovascular functioning, and can also, to some extent, buffer the accumulation of negative emotional experiences such as anxiety and depression (28, 32, 33). However, in contrast to this broad practical picture, the measurement and evaluation of older adults' participation in Health Qigong as a specific behavior remains relatively underdeveloped (34). Existing studies have mostly used single-dimensional indicators such as exercise frequency or duration as rough substitutes, and there is still a lack of standardized assessment tools that can accurately characterize the multidimensional participation status of older practitioners in terms of depth of regularity of practice, quality of attentional involvement, and persistence over time (35–39). In this study, Health Qigong engagement (HQE) refers to how regularly older adults practice, how closely they coordinate movement, breathing, and mental focus, and how independently they persist over time. Health Qigong is also commonly practiced in community groups, where repeated interaction with peers and instructors may provide companionship, mutual recognition, and a sense of belonging (17). This gap at the measurement level objectively constitutes a methodological constraint on further exploring the internal association between HQE and variables related to mental health among older adults (40).

When one form of physical activity can become so deeply embedded in the daily lives of older adults and continuously gain their emotional identification, exploring through what psychological pathways such engagement becomes connected with PWB becomes a theoretical issue that urgently needs to be addressed (13, 41–43). Self-determination theory is a motivational framework proposing that sustained engagement and psychological wellbeing are supported when the needs for autonomy, competence, and relatedness are met (44). In Health Qigong practice, autonomy is reflected in older adults' voluntary choice and self-regulation of practice; competence develops through the gradual mastery of movements and the coordination of body, breath, and mind; and relatedness is supported through regular interaction and shared practice with peers (45). The satisfaction of these needs may facilitate the internalization of Health Qigong practice and link sustained engagement to broader dimensions of PWB. According to the broaden-and-build theory of positive emotions, the positive emotions experienced by individuals in daily life contexts have a dual adaptive function of broadening momentary thought-action repertoires and building enduring personal resources (46–48). The soothing rhythm and inward-focused state created during Health Qigong practice provide older participants with a psychological space temporarily detached from daily trivial affairs (30). This experience helps buffer the inertial accumulation of negative emotions and provides a cognitive basis for the positive self-evaluation on which PWB depends (49). At the same time, mind-body interaction theory also suggests that there is a bidirectional nonverbal pathway linking the degree of external bodily extension and the level of internal psychological relaxation (50, 51). The bodily posture adjustments required in Health Qigong, such as sinking the shoulders and elbows and slightly hollowing the chest while lifting the back, are accompanied by practitioners' awareness of bodily boundaries and inner tension (52, 53). This gentle attention to and active regulation of bodily states may, to some extent, enhance older adults' acceptance of their own physical abilities (54). Such positive awareness of one's own body is precisely central to the dimensions of self-acceptance and environmental mastery in the construct of PWB (55, 56). Based on the above theoretical reasoning, and drawing on a cross-sectional research design, the present study proposes the following hypothesis: there is a significant positive correlation between the level of HQE and PWB among older adults (57, 58).

### 1.2 The relationship among HQE, RES, and PWB

The mechanism linking HQE and PWB may involve a more complex transmission pathway rather than a simple direct correspondence (30). To deepen the understanding of this relationship, it is necessary to introduce RES, a core construct reflecting an individual's ability to adapt to stress and recover from adversity, and to examine whether it plays a mediating role between the two (34, 59, 60). RES refers to the ability or process through which individuals can effectively adjust and maintain relatively stable psychological functioning when facing adversity, trauma, or major life stress (49, 61). For older adults, who are confronted with multiple pressures such as physical decline and social role transition, this quality has particularly prominent protective significance for maintaining quality of life in later years (62, 63). Within self-determination theory, the need-supportive experiences generated during Health Qigong are expected to produce internalized regulatory resources before they are reflected in broader wellbeing outcomes (44). RES is therefore positioned as the mediator because it represents the adaptive capacity through which the autonomy, competence, and relatedness experienced in practice can be generalized to coping with age-related stress. RES is not conceptualized as a product of physical movement alone. Repeated mastery experiences, coping with practice difficulties, and encouragement or modeling within group practice may also support confidence and adaptive coping. On the one hand, from a theoretical perspective, the stress-buffering theory suggests that repeatedly experiencing mind-body regulation training in low-intensity and controllable situations helps improve individuals' psychological tolerance threshold for coping with subsequent stressful events (64–66). The continuous regulation of breathing and attention during Health Qigong practice can be regarded as such a gentle form of mind-body regulation practice (67, 68). Therefore, it is reasonable to infer that older adults with higher levels of HQE may possess more stable RES (29). On the other hand, as an important internal resource for coping with difficulties, the promoting effect of RES on PWB has been widely supported by cross-sectional studies (69, 70). Older adults with higher levels of RES are often better able to maintain a positive self-evaluation and a sense of meaning in life under stressful circumstances (71). Based on the above logical chain, the present study proposes the following hypothesis: RES plays a mediating role between HQE and PWB among older adults (71, 72).

### 1.3 The moderating role of PSS

Perceived social support (PSS) refers to the individual's subjective experience and belief regarding the availability of emotional care, informational assistance, and companionship from social networks such as family members, peers, and the community (73, 74). The present study focuses on this perceived availability of support rather than on the broader structure and resources of social networks. For older adults, whose range of social interaction is relatively narrowed, this inner sense of confidence in the availability of support constitutes an important external resource for maintaining mental health (75). From the perspective of self-determination theory, PSS is not conceptualized as a consequence of HQE, but as a social-contextual condition that can support or constrain the satisfaction of basic psychological needs. Encouragement, companionship, and constructive feedback from family members, peers, and instructors may strengthen relatedness, affirm competence, and preserve the voluntary character of participation (44). The strength of the association between HQE and RES may vary depending on the level of PSS (60). Under conditions of high PSS, older practitioners may not only directly obtain the benefits of physical and psychological adjustment from Health Qigong practice, but may also consolidate the continuity of practice through encouragement and emotional companionship from significant others, thereby making the positive association between HQE and RES closer (76, 77). In contrast, under conditions of low PSS, individuals may lack reliable social connections (73). Even if they maintain regular HQE, the positive physical and psychological resources they accumulate may be less likely to be effectively transformed into stable RES because of insufficient external reinforcement, and the positive association between the two may therefore be weakened (49, 78). Accordingly, the present study proposes the following hypothesis: PSS plays a moderating role in the relationship between HQE and RES, such that the positive association between HQE and RES is more significant under conditions of higher PSS (72, 79).

### 1.4 Research objectives and hypotheses

Although sport engagement has been associated with resilience and wellbeing in younger populations (80, 81), whether this relationship extends to Health Qigong among older adults remains unclear, particularly in the absence of a context-specific instrument that captures the coordinated regulation of body, breath, and mind.

Based on the above, the present study aims to develop a Health Qigong Engagement Scale applicable to Chinese older adults, and on this basis to construct a moderated mediation model to systematically examine the relationships among HQE, RES, PSS, and PWB. Specifically, the present study intends to test the following hypotheses:

- H1: There is a significant positive correlation between HQE and PWB among older adults;
- H2: RES plays a mediating role between HQE and PWB;
- H3: PSS plays a moderating role in the relationship between HQE and RES, such that the positive association between HQE and RES is more significant under conditions of higher PSS. The hypothesized model of the present study is shown in Figure 1.

**Figure 1:**
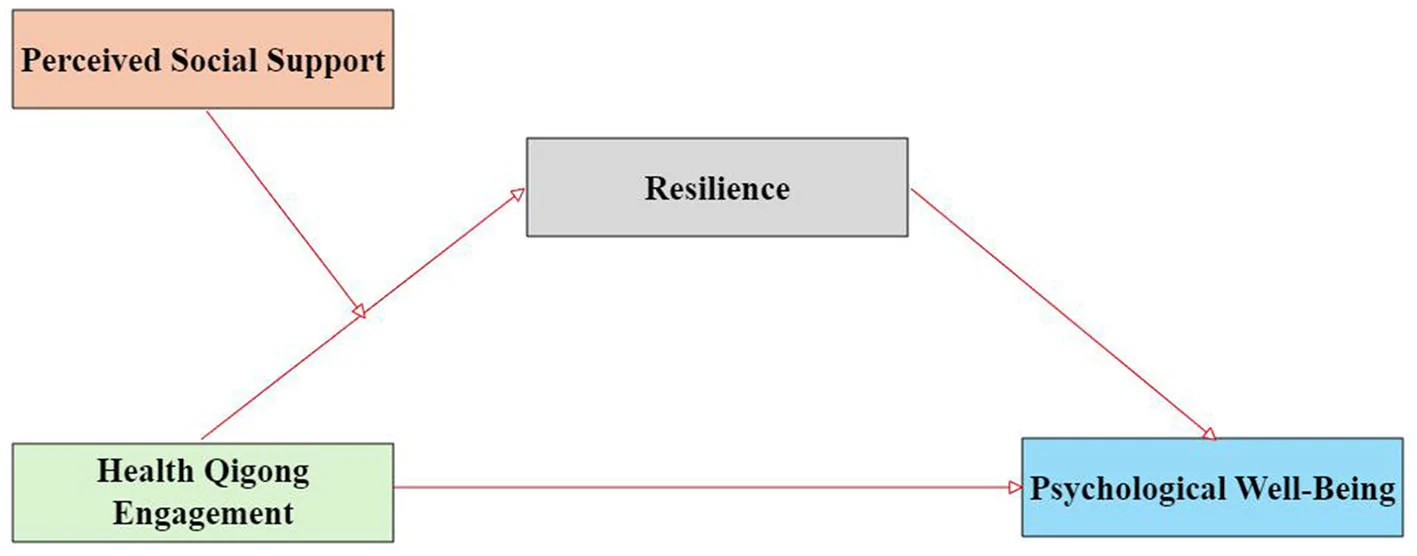
Hypothesized model of the present study.

## 2 Positive aging paradigms and theoretical framework

### 2.1 Conceptual foundations and distinctions among positive aging paradigms

Aging research has progressively moved beyond accounts of later life defined primarily by decline, dependency, and disease toward perspectives that also recognize retained capacities, adaptation, participation, meaning, and wellbeing (82). Within this broader shift, a number of positive aging paradigms have been developed to describe what it means to age well. These paradigms share an appreciative orientation toward later life, but they are not synonymous because they emphasize different criteria, processes, and contexts of aging.

Successful aging has traditionally emphasized the maintenance of physical and cognitive functioning, a low burden of disease and disability, and continued engagement with life (83). Productive aging places greater emphasis on older adults' continuing contributions through socially valued roles and activities, including employment, volunteering, caregiving, and other forms of social contribution. Active aging broadens the focus from individual outcomes to the opportunities and conditions that enable older adults to remain engaged in physical, social, cultural, and community life, with health, participation, and security forming interconnected domains (84). Healthy aging further shifts attention toward functional ability and the interaction between individual capacities and the environments in which older people live (85). Positive aging adds a more explicitly psychological and experiential dimension by emphasizing strengths, adaptation, meaning, personal growth, and psychological wellbeing in later life (86).

Considered together, these perspectives show that aging well cannot be reduced to a single outcome. They range from relatively outcome-oriented formulations of maintained functioning or social contribution to broader process- and context-oriented accounts of participation, person–environment interaction, and positive psychological functioning. For the present study, active, healthy, and positive aging provide the closest conceptual foundation because the focal issue is not whether older adults meet an idealized standard of disease-free or highly productive aging, but how sustained participation in a meaningful activity is situated within personal adaptive resources, supportive social conditions, and psychological wellbeing. These three perspectives can therefore be understood as complementary dimensions of a broader aging-well process rather than as separate explanations.

### 2.2 An integrated framework for health Qigong engagement and psychological wellbeing

Health Qigong provides a culturally grounded context in which this integrated aging process can be examined. Its relevance to active aging lies not simply in being a form of physical exercise, but in providing an activity that can be voluntarily sustained, incorporated into everyday routines, and practiced in individual or community settings (87). The construct of Health Qigong engagement (HQE) therefore extends the idea of participation beyond a binary distinction between being active and inactive: it captures continuity of practice, focused coordination of movement, breathing, and attention, and autonomous persistence over time. In this sense, HQE reflects the quality with which older adults remain engaged in a meaningful later-life activity.

The healthy aging perspective adds an important relational dimension to this participation. Maintaining valued activities in later life depends not only on the activity itself, but also on the resources available to the person and the conditions of the surrounding environment (88). RES is relevant here as an adaptive psychological resource that may help older adults respond flexibly to age-related and everyday challenges, while PSS represents the perceived availability of interpersonal resources from family members, peers, and other significant people. These constructs are not treated as direct equivalents of functional ability or environment; rather, they capture psychological and social resources that are conceptually consistent with the broader healthy-aging emphasis on the interaction between personal capacities and supportive contexts.

The positive aging perspective provides the psychological endpoint that gives this process broader significance. PWB concerns whether older adults continue to experience self-acceptance, purpose in life, personal growth, autonomy, environmental mastery, and positive relationships despite the changes and constraints that may accompany later life. Accordingly, the association between HQE and PWB can be interpreted within a broader process in which meaningful participation is embedded in personal and social resources and is linked with positive psychological functioning. Self-determination theory operates at a more proximal motivational level within this framework: autonomy, competence, and relatedness help explain why participation in Health Qigong may become internalized and sustained, whereas the aging paradigms clarify the broader significance of such engagement for aging well.

This integrated framework gives the empirical model a coherent aging-related interpretation. HQE, RES, PSS, and PWB are viewed as interconnected elements of meaningful participation, adaptive resources, social context, and positive functioning rather than as variables mechanically assigned to separate paradigms. Examining their associations therefore provides a culturally specific example of how positive aging can be studied through a traditional Chinese mind–body practice. It also broadens the study of later-life participation by focusing on the quality and persistence of engagement rather than exercise frequency alone, while recognizing that positive psychological functioning is embedded in both personal and interpersonal resources.

## 3 Methods

### 3.1 Participants and procedures

From January to June 2025, eligible older Health Qigong practitioners were recruited through participating Health Qigong associations and universities for older adults in six cities: Taiyuan and Changzhi in Shanxi Province, Xi'an and Ankang in Shaanxi Province, and Zhengzhou and Shangqiu in Henan Province. The selection of these six cities was mainly based on the following considerations: these areas are all representative regions in central and northwestern China where Health Qigong activities are relatively active, and the local organizational networks of Health Qigong associations and universities for older adults are relatively well established, which could provide a more standardized sampling frame for sample selection. At the same time, the multi-province sampling approach combining provincial capitals and prefecture-level cities helped, to a certain extent, enhance the diversity of the sample in terms of geographical distribution and socioeconomic background, thereby improving the external validity of the study findings. During the study period, a total of 1,259 paper questionnaires were distributed. According to the preset exclusion criteria, 86 invalid questionnaires were excluded due to incomplete responses, regular response patterns, and failure on lie-detection items, and 1,173 valid questionnaires were finally collected, with an effective response rate of 93.17%. For the small amount of missing data in the valid questionnaires, the multiple imputation by chained equations method was used for processing, and the imputation procedure was completed using the mice package in the R environment. A total of 1,173 older participants were ultimately included in the analysis, including 470 men (40.10%) and 703 women (59.90%). In terms of age structure, 616 were aged 60 to 70 years (52.50%), 371 were aged 71–80 years (31.60%), and 186 were aged over 80 years (15.90%). In terms of marital status, 834 had a spouse (71.10%) and 339 did not have a spouse (28.90%). In terms of educational level, 149 were illiterate or semi-illiterate (12.70%), 356 had a primary school education (30.30%), 454 had a middle school education (38.70%), and 214 had a high school education or above (18.20%). In terms of annual income, 706 earned less than 7,500 RMB (60.20%), and 467 earned more than 7,500 RMB (39.80%). Detailed information is shown in Table 1.

**Table 1:** Distribution of basic sample characteristics (*N* = 1,173).

Variable	Category	Count	Column *N* %
Gender	Male	470	40.1%
	Female	703	59.9%
Age	60–70 years	616	52.5%
	71–80 years	371	31.6%
	Over 80 years	186	15.9%
Marital status	With a spouse	834	71.1%
	Without a spouse	339	28.9%
Educational level	Illiterate or semi-illiterate	149	12.7%
	Primary school	356	30.3%
	Middle school	454	38.7%
	High school or above	214	18.2%
Annual income	Less than 7,500 RMB	706	60.2%
	More than 7,500 RMB	467	39.8%

### 3.2 Measures

#### 3.2.1 PWB scale

Psychological wellbeing (PWB) was assessed using an 18-item Chinese scale based on Li (89). The items measure six related dimensions: autonomy, environmental mastery, personal growth, positive relations with others, purpose in life, and self-acceptance. Responses range from 1 (strongly disagree) to 6 (strongly agree). The mean item score was used, with higher scores indicating greater PWB. In the present sample, Cronbach's α was 0.837, and confirmatory factor analysis supported the six-factor structure (χ^2^/df = 1.070, RMSEA = 0.008, SRMR = 0.018, CFI = 0.998, TLI = 0.998).

#### 3.2.2 RES scale

Resilience (RES) was assessed using the 25-item Connor-Davidson Resilience Scale developed by Connor and Davidson (90) and adapted into Chinese by Yu and Zhang (91). The items jointly assess the capacity to adapt to stress and recover from adversity. Responses range from 1 (not true at all) to 5 (true nearly all the time). The mean item score was used, with higher scores indicating greater RES. In the present sample, Cronbach's α was 0.918, and confirmatory factor analysis supported the measurement structure (χ^2^/df = 1.272, RMSEA = 0.015, SRMR = 0.017, CFI = 0.995, TLI = 0.994).

#### 3.2.3 PSS scale

Perceived social support (PSS) was assessed using a 12-item scale covering support from family, friends, and other important people (92). Responses range from 1 (very strongly disagree) to 7 (very strongly agree). The mean item score was used, with higher scores indicating greater perceived availability of social support. In the present sample, Cronbach's α was 0.895, and confirmatory factor analysis supported the three-factor structure (χ^2^/df = 2.035, RMSEA = 0.030, SRMR = 0.015, CFI = 0.994, TLI = 0.992).

#### 3.2.4 Self-developed HQE scale

In developing the HQE Scale, the present study adopted self-determination theory as the core theoretical framework. This theory suggests that the sustainability of individual behavior and the quality of engagement largely depend on the extent to which three basic psychological needs—autonomy, competence, and relatedness—are satisfied. This theoretical perspective provides an appropriate explanatory path for understanding the multidimensional structure of HQE among older adults. Guided by this theoretical orientation and in combination with the unique characteristics of Health Qigong, the present study defined HQE as a construct consisting of three interrelated dimensions. The first dimension is continuous behavioral engagement, which focuses on the frequency and temporal stability of older adults' participation in Health Qigong practice, reflecting the individual's external behavioral performance in terms of practice frequency, duration of each session, and long-term regularity, and constituting the foundational dimension for measuring the level of engagement. The second dimension is normative focused engagement. Given that the core feature distinguishing Health Qigong from general physical activity lies in its emphasis on the coordinated operation of regulating the body, regulating the breath, and regulating the mind, this dimension is intended to characterize the depth of engagement of older practitioners in terms of adherence to movement standards, awareness of the coordination between breathing and movement, and the focused state of inward attention, thereby reflecting the qualitative connotation of HQE. The third dimension is autonomous persistent engagement. This dimension goes beyond the mere level of behavioral execution and points to the individual's inner driving force and tendency toward active self-regulation during participation in Health Qigong, as reflected in older practitioners' ability to arrange practice autonomously in the absence of supervision and their willingness to persist in the face of objective obstacles such as weather changes and physical fatigue. These three dimensions together constitute a more comprehensive and refined measurement framework for assessing the level of HQE among older adults.

At the operational level of scale development, the present study adopted an analytical approach oriented by grounded theory. Qualitative data were obtained through semi-structured interviews, and the dimensional structure and measurement items were extracted through a three-level coding procedure. In the preliminary stage, the research team first conducted a literature review and information search around older adults' participation in Health Qigong and initially identified the core directions of the interview outline. Subsequently, 15 older adults with regular experience in Health Qigong practice were selected as interview participants, and in-depth interviews were conducted around their participation frequency, practice habits, attention to movements, awareness of breathing coordination, autonomy in practice, and persistence experience. In the coding analysis stage, open coding was first conducted to conceptualize and extract the original interview statements. For example, one participant stated, “Every morning after taking my grandson to school, I go to the park and do Baduanjin twice. When it rains, I practice in the living room at home. Anyway, if I don't practice, it feels like something is missing.” From this, initial concepts such as “practicing at a fixed time every day,” “maintaining practice despite space limitations,” and “forming a sense of psychological dependence” were extracted. Another participant stated, “When practicing, I need to calm my mind and let the movements go together with the breathing. I can't just imitate the appearance.” From this, initial categories such as “seeking mental calmness,” “emphasizing synchronization of breathing and movement,” and “distinguishing outward imitation from inner essence” were extracted. Axial coding was then conducted to integrate the scattered initial concepts into more overarching categories. For example, concepts such as “stable practice frequency,” “guaranteed practice duration,” and “long-term uninterrupted persistence” were grouped into the core category of “behavioral continuity”; concepts such as “self-checking movement standards,” “conscious coordination of breathing rhythm,” and “attention following movements” were grouped into the category of “practice normativity”; and concepts such as “actively practicing without supervision,” “self-adjusting when encountering difficulties,” and “actively resuming practice after interruption” were grouped into the category of “autonomous regulation.” Finally, selective coding was conducted, and the three core dimensions of “continuous behavioral engagement,” “normative focused engagement,” and “autonomous persistent engagement” were established as the main structural components of the scale construct. Regarding the test of theoretical saturation, after completing the coding of data from the first 13 interviewees, the research team reserved two interview records for saturation testing. Comparative analysis showed that no new conceptual categories or dimensional relationships were identified from the reserved materials, indicating that the existing coding system had already sufficiently covered the core characteristics of HQE among older adults, and that the theoretical saturation had reached an acceptable level.

After completing the grounded theory-based dimensional construction and the formulation of the initial item pool, the present study further adopted a two-round Delphi expert consultation method to evaluate the content validity and screen the items of the first draft of the scale, so as to ensure the scientific soundness and cultural appropriateness of the measurement tool. In terms of expert selection, the research team invited a total of 12 experts from relevant fields to form the consultation panel, including four senior coaches or referees in the field of Health Qigong, three scholars in geriatric psychology, three researchers in sports measurement and evaluation, and two experts in geriatric social work. The experts had an average of 14.6 years of professional experience, and nine held associate senior or above professional titles. In the first round of consultation, the research team distributed an evaluation questionnaire containing dimension definitions and the initial item pool to all experts, inviting them to rate each item in terms of the degree of match between the item and the dimension, clarity of expression, and cultural appropriateness. A 5-point Likert scale was used for scoring, and an open-ended section for revision suggestions was also provided. After the first round of consultation, the items were reviewed according to the preset screening criteria: items with a mean above 4.0 and a coefficient of variation below 0.25 were retained, while items that failed to meet the criteria were deleted or substantively revised according to expert suggestions. In the first round, the initial item pool consisted of 21 items. The item “I pay attention to matching the music rhythm during Health Qigong practice” was deleted because its coefficient of variation was high and several experts pointed out that music rhythm is not a core element of Health Qigong practice. The item “I record my reflections after practice” was deleted because its mean score did not meet the standard and experts believed that it did not conform to the behavioral habits of most older practitioners. At the same time, according to expert feedback, two new items were added. One was “When practicing Health Qigong, I try to eliminate the interference of distracting thoughts,” which was used to strengthen the connotation of attentional regulation in the dimension of normative focused engagement. The other was “When I feel physically uncomfortable, I adjust the range of movement accordingly instead of giving up directly,” which was used to enrich the characteristic of flexibility in coping with obstacles in the dimension of autonomous persistent engagement. After the first-round revision, a second-round evaluation questionnaire containing 20 items was formed. In the second round of expert consultation, the mean score of all items exceeded 4.2, and all coefficients of variation were below 0.20. Kendall's W coefficient of coordination among expert opinions increased significantly compared with the first round and reached statistical significance, indicating that expert consensus had become more consistent. After screening and revision through the two-round Delphi procedure, a preliminary HQE Scale containing 18 items was finally formed, laying the content validity foundation for subsequent item analysis and exploratory factor analysis.

After completing the expert evaluation of item content validity, the present study further conducted quantitative screening and structural validation of the preliminary scale through item analysis, exploratory factor analysis, and confirmatory factor analysis. In the item analysis stage, the critical ratio method and the item-total correlation method were used to test the discrimination of each item. The results showed that the decision values of all 18 items were significantly higher than the critical standard of 3.0 (t values ranged from 18.373 to 25.652, *p* < 0.001), and the correlation coefficients between each item and the total score of its corresponding dimension were all greater than the preset threshold of 0.40 (r values ranged from 0.668 to 0.722, *p* < 0.001), indicating that the items had good discrimination and homogeneity and were therefore all retained. Subsequently, the valid sample was randomly split into two halves, one for exploratory factor analysis and the other for confirmatory factor analysis. Exploratory factor analysis was conducted using principal component analysis and varimax orthogonal rotation. The Kaiser-Meyer-Olkin value was 0.938, and the approximate chi-square value of Bartlett's test of sphericity was 6462.500 (df = 153, *p* < 0.001), indicating that the data were suitable for factor analysis. According to the criterion of eigenvalues greater than 1, three common factors were extracted, with a cumulative variance explained of 86.95%. The factor loadings of all items on their respective factors ranged from 0.847 to 0.908, all of which were higher than the preset critical value of 0.55, and no excessive cross-factor loadings occurred. The rotated factor structure was fully consistent with the three-dimensional construct established in the earlier qualitative study. On this basis, confirmatory factor analysis was conducted on the other half of the sample data to cross-validate the robustness of the factor structure. The results of confirmatory factor analysis showed that all fit indices of the three-factor model reached a good level (χ^2^/df = 1.965, RMSEA = 0.055, SRMR = 0.052, CFI = 0.980, TLI = 0.977), indicating that the three-dimensional structure of the scale was effectively supported at the statistical level. In terms of reliability testing, the overall Cronbach's α coefficient of the scale was 0.938, and the Cronbach's α coefficients of the three subdimensions—continuous behavioral engagement, normative focused engagement, and autonomous persistent engagement—were 0.956, 0.956, and 0.959, respectively, all of which were significantly higher than the acceptable threshold of 0.70, indicating that the measurement tool had relatively satisfactory internal consistency reliability. Detailed statistical indices and figures for the above quantitative analysis process are shown in Appendix Tables 1–4 and Appendix Figure 1.

### 3.3 Statistical analysis

The present study employed multiple statistical software packages to collaboratively complete data processing and analysis. For qualitative data, the coding and category extraction of interview transcripts were conducted using NVivo 14, in order to systematically advance the processes of open coding, axial coding, and selective coding under the grounded theory approach. Quantitative data analysis mainly relied on SPSS 27.0 and AMOS 24.0. Specifically, SPSS 27.0 was used for descriptive statistical analysis (including means, standard deviations, skewness, and kurtosis), critical ratio tests and item-total correlation analysis in item analysis, exploratory factor analysis, internal consistency reliability testing, Pearson correlation analysis among the main variables, and Harman's single-factor test for common method bias. Confirmatory factor analysis was conducted using AMOS 24.0 to construct measurement models and evaluate the goodness of fit of the factor structures of each scale. In addition, hypothesis testing of the mediating effect and moderating effect was performed using the PROCESS 3.5 macro installed in SPSS 27.0, and path coefficients and confidence intervals were estimated based on the Bootstrap method recommended by Hayes.

## 4 Results

### 4.1 Common method bias test

Given that all data in the present study were derived from self-reports provided by older participants, there was a possibility that common method bias might potentially interfere with the study conclusions. Therefore, Harman's single-factor test was conducted for statistical examination. Specifically, all measurement items in the questionnaire were entered simultaneously into an exploratory factor analysis, and the unrotated factor solution was examined without factor rotation. The results showed that a total of 10 independent common factors with eigenvalues greater than 1 were extracted, among which the first common factor accounted for 17.38% of the total variance. This value was significantly lower than the empirical critical threshold of 40%, indicating that a single factor did not explain the majority of the variance. Accordingly, it can be inferred that there was no serious common method bias in the data collected in the present study that would threaten the validity of the conclusions, and the subsequent statistical analysis results regarding the relationships among variables have a certain degree of credibility.

### 4.2 Descriptive statistics and correlation analysis

Descriptive statistics showed that the main variables were approximately normally distributed, with skewness values ranging from −0.060 to 0.053 and kurtosis values ranging from −0.940 to −0.292. Pearson correlation analysis was conducted on the main study variables to preliminarily examine the associations among HQE, RES, PSS, and PWB. The correlation matrix showed that HQE was significantly positively correlated with PWB, RES, and PSS, respectively. RES was also significantly positively associated with both PWB and PSS. In addition, PSS was significantly positively correlated with PWB. The means, standard deviations, skewness, kurtosis, correlation coefficients and significance levels among all variables are presented in Table 2.

**Table 2:** Correlation coefficients and descriptive statistics of all variables (*N* = 1,173).

Variable	*M*	SD	Skewness	Kurtosis	HQE	PWB	RES	PSS
HQE	3.147	0.865	0.053	−0.583	1	–	–	–
PWB	3.501	0.880	−0.060	−0.458	0.164^***^	1	–	–
RES	3.132	0.827	−0.023	−0.292	0.453^***^	0.160^***^	1	–
PSS	3.892	1.423	−0.038	−0.940	0.331^***^	0.105^***^	0.345^***^	1

^***^p < 0.001. *M*, mean; SD, standard deviation; HQE, Health Qigong Engagement; PWB, Psychological wellbeing; RES, resilience; PSS, perceived social support.

### 4.3 Mediation analysis

The present study used hierarchical regression analysis and the Bootstrap method to examine the hypothesized indirect association through RES. After controlling for demographic variables including gender, age, marital status, educational level, and annual income, the association between HQE and RES was first examined. The results showed that HQE was significantly positively associated with RES. Then, HQE and RES were entered simultaneously into the regression model predicting PWB. The results indicated that the regression coefficient of RES on PWB reached a significant level, and although the direct effect of HQE on PWB was reduced, it remained statistically significant. Furthermore, the bias-corrected percentile Bootstrap method was used to estimate the confidence interval of the indirect effect. After 5,000 repeated samples, the results showed that the total effect was 0.176, of which the direct effect was 0.127 and the indirect effect was 0.049. The 95% confidence interval of the indirect effect did not include zero, indicating a statistically significant cross-sectional indirect effect. Effect decomposition showed that the direct effect accounted for 72.03% of the total effect, whereas the indirect effect accounted for 27.97% of the total effect. These results support the research hypothesis that RES plays a partial mediating role between HQE and PWB among older adults. Detailed regression parameters and effect decomposition are shown in Tables 3, 4.

**Table 3:** Regression results for the mediating effect of RES.

Dependent variable	Independent variable	*R*	*R^2^*	*F*	β	*t*
RES	HQE	0.465	0.216	53.619	0.431	16.177
	Gender				0.009	0.172
	Age				0.116	1.185
	Marital status				0.099	1.648
	Education level				−0.002	−0.087
	Annual income				−0.066	−1.242
PWB	HQE	0.201	0.041	7.032	0.127	3.899
	RES				0.115	3.534
	Gender				0.023	0.382
	Age				−0.054	−1.319
	Marital status				−0.083	−1.253
	Education level				0.030	0.947
	Annual income				0.005	0.092

HQE, Health Qigong Engagement; PWB, psychological wellbeing; RES, resilience; PSS, perceived social support.

**Table 4:** Bootstrap decomposition of the mediating effect.

Effect	Effect size	Bootstrap standard error	95% lower	95% upper	Effect size (%)
Total	0.176	0.030	0.118	0.235	–
Direct	0.127	0.033	0.063	0.191	72.03%
Indirect	0.049	0.012	0.027	0.074	27.97%

### 4.4 Moderation analysis

The present study further examined the moderating role of PSS in the relationship between HQE and RES. After controlling for demographic variables including gender, age, marital status, educational level, and annual income, HQE, PSS, and their interaction term were simultaneously entered into the regression model to predict RES. The results showed (Table 5) that the regression coefficient of the interaction term between HQE and PSS on RES reached a significant level (β = 0.062, 95% CI [0.010, 0.114]), indicating that PSS had a positive moderating effect on the relationship between the two. To further reveal the specific pattern of the moderating effect, a simple slope test was conducted. The results showed that under a low level of PSS, the effect value of HQE on RES was 0.300 (*p* < 0.001); under a medium level of PSS, the effect value was 0.362 (*p* < 0.001); and under a high level of PSS, the effect value increased to 0.424 (*p* < 0.001). These results indicate that, as the level of PSS increased, the positive association between HQE and RES showed an increasing trend, and the research hypothesis was supported. The moderation effect plot is shown in Figure 2.

**Table 5:** Regression results for the moderating effect of PSS.

Dependent variable	Predictor variable	β	95% lower	95% upper	*T*
RES	HQE	0.362	0.308	0.416	13.228
	PSS	0.219	0.166	0.271	8.191
	HQE × PSS	0.062	0.010	0.114	2.336
	Gender	0.004	−0.097	0.106	0.082
	Age	0.112	0.043	0.182	3.171
	Marital status	0.103	−0.012	0.217	1.758
	Education level	−0.009	−0.063	0.044	−0.339
	Annual income	−0.056	−0.158	0.046	−1.080

HQE, Health Qigong Engagement; RES, resilience; PSS, perceived social support.

**Figure 2:**
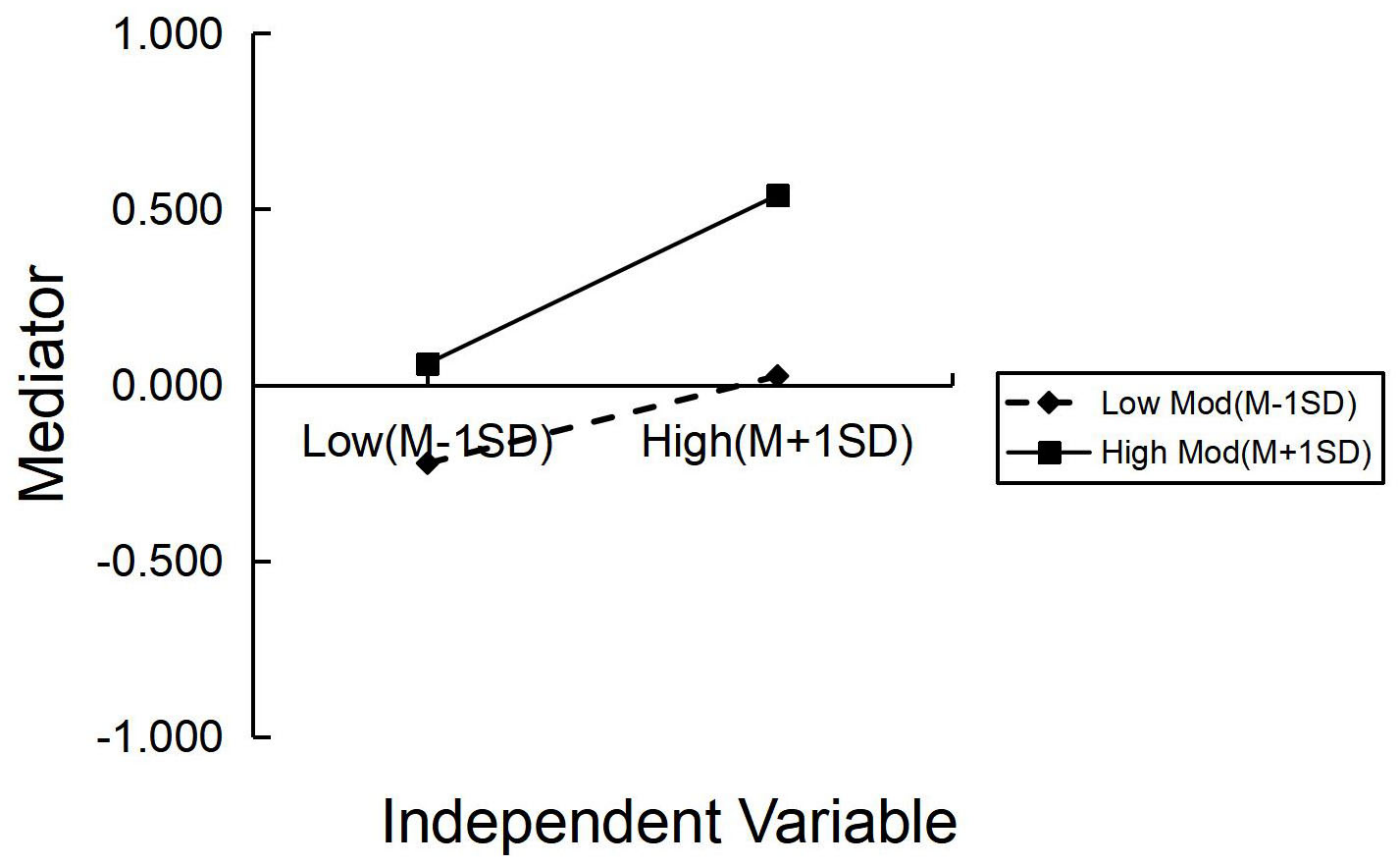
Simple slope plot of the moderating effect of PSS on the relationship between HQE and RES.

## 5 Discussion

### 5.1 Structural interpretation and theoretical implications of the HQE scale

The present study developed the Health Qigong Engagement Scale for Chinese Older Adults. Through standardized procedures including qualitative coding, Delphi consultation, and quantitative testing (87), three core dimensions were ultimately established: continuous behavioral engagement, normative focused engagement, and autonomous persistent engagement (93). At the theoretical level, this three-dimensional structure effectively corresponds to the basic propositions of self-determination theory, namely that the depth of an individual's behavioral involvement depends on the coordinated fulfillment of external behavioral habits, internal competence experience, and autonomous self-regulation willingness. The dimension of continuous behavioral engagement reflects the external behavioral manifestations of older adults' HQE in terms of practice frequency, duration, and regularity, and serves as a foundational indicator for measuring the level of engagement (94). Its significance lies in capturing whether older adults have stably embedded Health Qigong into the rhythm of their daily lives. The dimension of normative focused engagement highlights the project-specific characteristics that distinguish Health Qigong from general physical activity (95). Its emphasis on adherence to movement standards, coordination of breathing rhythm, and focused inward attention reflects older practitioners' pursuit of practice quality and their understanding of the inner connotations of the exercises, thus going beyond a superficial description of mere attendance or non-attendance (96). The dimension of autonomous persistent engagement further touches on the level of the internal driving force behind participation behavior, as manifested in older adults' willingness to arrange practice proactively in the absence of external supervision, as well as their tendency to flexibly adjust and continue practicing when facing objective obstacles such as weather changes and physical discomfort (97, 98). This reflects the degree to which individuals have internalized identification with Health Qigong activity (99). The establishment of these three-dimensional constructs not only provides a progressive evaluative framework for HQE, ranging from behavioral frequency to cognitive engagement and then to volitional maintenance, but also compensates for the measurement limitation of previous studies, which often used a single frequency indicator as a rough substitute for engagement level, and provides a localized measurement tool with stronger reliability and validity for subsequent research on the relationships between HQE and variables such as RES and PWB (67). In applied settings, the overall HQE score provides a summary of engagement, while the three dimension scores can indicate whether lower engagement is mainly reflected in irregular practice, limited attentional involvement, or difficulty maintaining self-directed practice. This distinction may help researchers and community instructors provide more targeted practice guidance.

### 5.2 The association between HQE and PWB

Based on cross-sectional data, the present study found a significant positive association between the level of HQE and PWB among older adults (100). This finding provides empirical reference for understanding the mind-body relationship within the daily exercise context of Chinese older adults (101). In typical settings where older adults practice Health Qigong, such as morning exercise spots in parks and community activity rooms, the soothing rhythm and group practice atmosphere created by Health Qigong together constitute a transitional space distinct from household chores and life stress (102). In this specific context, older practitioners temporarily shift their attention away from daily worries and toward inner awareness of bodily states through the performance of slow, smooth, and rounded movements and the regulation of even and deep breathing (103). Such a shift in attentional focus helps buffer the daily accumulation of negative emotions (104). At the same time, Health Qigong practice is usually organized in the form of collective morning exercise (105). Older adults complete the exercises together with peers at relatively fixed times and locations (106). Repeated shared practice may provide companionship, mutual recognition, and a sense of belonging, even when interactions remain informal. These socio-emotional experiences may complement the effects of movement, breathing regulation, and attentional focus and may help reduce the sense of alienation associated with withdrawal from social roles (107). From the perspective of the broaden-and-build theory of positive emotions, the inner calmness and mild pleasure generated by the above practice context may not reach the level of intense emotional arousal, but their cumulative effect may, over a longer time scale, extend older adults' cognitive flexibility, enabling them to view changes and challenges in later life from a more positive perspective, and thereby present a more favorable state in core dimensions of PWB, such as self-acceptance and environmental mastery (108–110).

### 5.3 The mediating role of RES

The cross-sectional mediation model indicated a statistically significant indirect association between HQE and PWB through RES, and this finding provides a more process-oriented explanatory perspective for understanding how HQE is associated with positive psychological experience among older adults (111). From the perspective of the context in which HQE fosters RES, the daily practice process of Health Qigong itself constitutes a mild yet continuous training field for RES. This interpretation does not treat resilience as a product of physical movement alone. In group settings, observing peers, receiving guidance, and sharing routine difficulties may provide models of mastery, encouragement, and opportunities to practice adaptive coping (112). In the day-to-day practice of Health Qigong routines, older adults need to continuously overcome the discomfort caused by bodily stiffness, the sense of frustration caused by forgetting movements, and the irritability triggered by distraction. This repeated adjustment to and overcoming of physical and psychological states in a low-intensity and controllable context in fact provides cumulative training opportunities for the adversity adaptation and self-regulation abilities emphasized by RES (113, 114). In particular, for older practitioners who show strong performance in the dimensions of normative focused engagement and autonomous persistent engagement, their continuous pursuit of movement standards during practice and their active persistence in the face of disturbances such as weather changes and physical fatigue correspond structurally to the inner qualities required by RES, namely maintaining goal-directed behavior in difficult situations (115, 116). From the perspective of the effect of RES on PWB, older adults with higher levels of RES often demonstrate more flexible adjustment strategies when dealing with unavoidable stressful events in later life (117). When encountering negative life events such as physical decline, the death of relatives and friends, or intergenerational conflict, older adults with high RES are more likely to view them as temporary challenges that can be coped with rather than irreversible losses (118). This cognitive framework helps buffer the eroding effect of stressful events on PWB, enabling them to maintain affirmation of self-worth and a sense of life meaning even under adversity (119). Taken together, these two pathways suggest that HQE may first cultivate the resilient qualities of coping with difficulties and persisting toward goals in the micro-context of daily practice, and that these inner psychological resources accumulated through long-term practice may then be transformed into a protective buffer for maintaining PWB when older adults face the macro-level challenges of later life (120). The mediating pathway revealed in the present study extends the positive effect of HQE from the practice context itself to the broader field of adaptation in later life, and provides a more detailed theoretical picture for understanding the mechanism through which Health Qigong, as a traditional form of mind-body cultivation, contributes to the mental health of older adults (121).

### 5.4 The moderating role of PSS

The present study also found that PSS plays a moderating role in the relationship between HQE and RES, and that the strength of the positive association between HQE and RES varies according to the level of social support subjectively perceived by older adults (122). For older practitioners with a higher level of PSS, Health Qigong practice is usually not an isolated individual behavior, but rather a collective practice embedded in supportive interpersonal networks involving family understanding, peer companionship, and community care (123). In such contexts, when older adults encounter difficulties in mastering movements or experience wavering persistence during practice, supportive interactions such as verbal affirmation from family members, experience sharing from peers, or patient guidance from instructors can provide emotional comfort and strengthen belief beyond the practice itself, thereby allowing the effect of Health Qigong practice on the cultivation of RES to be more fully manifested (124). In contrast, among older adults with a lower level of PSS, Health Qigong practice is more likely to appear as an independent activity sustained mainly by personal will. Although such practitioners may likewise obtain physical and psychological adjustment benefits from practice, the absence of perceivable interpersonal support as an external reinforcing resource makes them more likely to fall into the difficulty of coping alone when facing practice obstacles or life troubles, and the efficiency with which the positive physical and psychological experiences accumulated during Health Qigong practice are transformed into stable RES may therefore be reduced (125). These findings suggest that the positive association between HQE and RES is not evenly distributed across all older practitioners. Rather, the level of supportiveness in their social ecological environment constitutes an important boundary condition constraining the strength of this association. Enhancing older adults' subjective perception of the availability of social support may help amplify the health-promoting effect of HQE (126).

### 5.5 Theoretical implications for positive aging paradigms

Viewed through the integrated aging framework, the findings extend the interpretation of Health Qigong beyond a conventional exercise–health association. The HQE Scale gives greater specificity to the active-aging emphasis on participation by showing that engagement can be characterized in terms of continuity, attentional quality, and self-directed persistence. This distinction is theoretically important because remaining active in later life is not only a matter of whether an activity occurs, but also of how meaningfully and sustainably it is incorporated into everyday life. Health Qigong therefore illustrates a culturally grounded form of later-life participation in which physical activity, focused mind–body involvement, and social embeddedness can coexist (84).

The associations involving RES and PSS also support a more contextual understanding of aging well. The cross-sectional indirect association through RES is consistent with the relevance of adaptive personal resources, while the stronger HQE–RES association at higher levels of PSS highlights the importance of the social environment within which adaptation occurs. These findings accord with the healthy-aging emphasis on the interplay between individual capacities and supportive contexts (85). At the same time, the positive association between HQE and PWB connects this participation-and-resource process with the positive-aging emphasis on meaning, self-acceptance, autonomy, growth, and positive relationships (86). Taken together, the findings demonstrate the value of examining aging well as an interconnected process rather than through a single criterion. They also extend this discussion to a Chinese cultural context in which a traditional mind–body practice may serve as an accessible setting for continued participation and positive psychological functioning. These interpretations remain associative because the study design does not establish temporal ordering or causality.

### 5.6 Practical implications

The HQE Scale may help researchers and community practitioners identify whether lower engagement is mainly reflected in irregular practice, limited coordination of movement, breathing, and attention, or difficulty maintaining self-directed practice. This information may support more targeted practice guidance, while family encouragement and peer companionship may also be considered when organizing community Health Qigong activities. In simple terms, participants who were more regularly, attentively, and independently engaged in Health Qigong also reported better psychological wellbeing, although the cross-sectional findings do not establish that Health Qigong practice caused these differences.

### 5.7 Limitations and future directions

Although the present study developed the Health Qigong Engagement Scale for Chinese Older Adults based on standardized procedures, and on this basis constructed a moderated mediation model to examine the mechanism linking HQE and PWB, there are still several limitations that need to be addressed in future research (59, 126). First, the present study adopted a cross-sectional design, and HQE, RES, and PWB were measured concurrently. Consequently, the temporal ordering required for mediation cannot be established. Cross-sectional estimates of indirect effects may differ substantially from longitudinal mediation parameters and can be biased under both complete and partial mediation models (127). The statistically significant indirect effect observed in this study should therefore be interpreted as an associative pattern consistent with, rather than evidence of, the proposed mediation process. Future research should use multi-wave longitudinal designs that separate the measurement of HQE, RES, and PWB over time, or randomized and intervention-based designs, to test temporal precedence and causal mechanisms. Second, the sample of the present study was drawn from Taiyuan and Changzhi (Shanxi Province), Xi'an and Ankang (Shaanxi Province), and Zhengzhou and Shangqiu (Henan Province). Although provincial capitals and prefecture-level cities were included to enhance regional representativeness, the geographical scope was still limited to midwest China, and the extent to which the findings can be generalized to older adults in other regions of the country remains to be tested. Future research may expand the geographical range of sampling and include older adult samples from East China, North China, Northwest China, and other regions with different socioeconomic and cultural backgrounds, so as to further verify the cross-regional applicability of the scale and the theoretical model. Third, all data in the present study were obtained through self-report questionnaires. Although the common method bias test did not indicate serious interference, subjective reports may still be affected by social desirability and recall bias. Future studies may attempt to introduce objective behavioral indicators as supplementary measures of HQE, such as practice attendance records, wearable-device data on practice duration, or third-party observational ratings of movement standardization, in order to improve the accuracy and multidimensionality of the measurement results (128). The present study also did not include objective physical health outcomes, such as balance, flexibility, cardiovascular function, or functional mobility. Therefore, the findings cannot determine whether higher HQE scores correspond to measurable physical health differences. Future studies should combine the HQE Scale with objective physical assessments to examine its broader practical relevance. Fourth, the present study focused on the mediating role of RES and the moderating role of PSS. However, the association between HQE and PWB may also involve other psychological mechanisms or contextual conditions that were not included in the present study (122). Future research may further explore the potential mediating effects of variables such as self-efficacy, mindfulness, and physical self-concept, as well as the moderating effects of environmental factors such as living arrangement and accessibility of community fitness facilities, so as to construct a more complete theoretical explanatory framework (129). Fifth, the present study did not directly assess motives for beginning or maintaining Health Qigong, the identities of people who introduced or encouraged practice, or memories of earlier shared practice. The findings therefore cannot determine whether emotional attachment to Health Qigong reflects autobiographical or relational memories. Future qualitative and mixed-method studies should examine these social and motivational processes directly. Sixth, although the HQE Scale developed in the present study demonstrated good reliability and validity among older adults, its applicability among younger and middle-aged practitioners or clinical rehabilitation populations remains unknown (130). Future research may further test the measurement invariance and applicability boundaries of this scale across groups of different age stages and health conditions, so as to expand its scope of application and instrumental value.

## 6 Conclusion

Based on a standardized scale development procedure, the present study developed the Health Qigong Engagement Scale for Chinese Older Adults, which includes three dimensions: continuous behavioral engagement, normative focused engagement, and autonomous persistent engagement. Psychometric testing showed that the scale has good reliability and validity among Chinese older adults, thus providing a localized measurement tool for subsequent related research. On this basis, the present study constructed a moderated mediation model to systematically examine the relationships among HQE, RES, PSS, and PWB. The results showed a significant positive association between HQE and PWB, a statistically significant cross-sectional indirect association through RES, and variation in the HQE–RES association according to PSS. These findings suggest a possible explanatory framework linking HQE, RES, PSS, and PWB, but they do not establish temporal or causal mediation. The study therefore provides a localized assessment tool and preliminary associative evidence that can inform future longitudinal and intervention-based research on Health Qigong and older adults' mental health. Within the broader positive aging framework, these findings also provide a culturally grounded example of how meaningful participation, adaptive personal resources, supportive social conditions, and positive psychological functioning can be considered together when examining aging well.

## Data Availability

The original contributions presented in the study are included in the article/Supplementary material, further inquiries can be directed to the corresponding authors.

## References

[1] Fang EF Fang Y Chen G Wang H-L Zhang J Wu C . Adapting health, economic and social policies to address population aging in China. Nat Aging. (2025) 5:2176–87. doi: 10.1038/s43587-025-00999-8 41174222

[2] Ji Q Yin M Li Y Zhou X. Exploring the influence path of high-rise residential environment on the mental health of the elderly. Sustain Cities Soc. (2023) 98:104808. doi: 10.1016/j.scs.2023.104808

[3] Guo J Abu Talib M Guo B Ren J Liu J. The mediating role of satisfaction with life and social interaction anxiety in the relationship between loneliness and regulatory emotional self-efficacy. Behav Sci. (2025) 15:392. doi: 10.3390/bs15030392 40150286 PMC11939567

[4] Komatsu H Niimura H Yagasaki K. Process of inner change in advanced age: a qualitative study of older adults in their early 90 s. BMC Geriatr. (2022) 22:945. doi: 10.1186/s12877-022-03665-5 36482303 PMC9733006

[5] Upasen R Saengpanya W Awae W Prasitvej P Stithyudhakarn S. Understanding how older adults sustain mental well-being when facing challenging in later life. Acta Psychol. (2025) 259:105293. doi: 10.1016/j.actpsy.2025.105293 40680646

[6] Rosdiana S Junaidi J Anwar SM Sahrir S. Analysis of the role of spiritual well-being on ethical judgment among accounting students. Nominal Barom Ris Akunt dan Manaj. (2025) 14:25–41. doi: 10.21831/nominal.v14i1.77030

[7] Martela F. Being as having, loving, and doing: a theory of human well-being. Pers Soc Psychol Rev. (2024) 28:372–97. doi: 10.1177/10888683241263634 39056545 PMC11500488

[8] Stelcer B Bendowska A Karkowska D Baum E. Supporting elderly patients in strengthening their personal and spiritual health resources. Front Psychol. (2023) 14:1237138. doi: 10.3389/fpsyg.2023.1237138 38023038 PMC10667473

[9] Aviisah MA Haisma HH Zotor FB Vogt TC. “Inner peace is the good life”: conceptualizations of subjective well-being among older adults aged 60 and over in rural Northern Ghana. J Happiness Stud. (2025) 26:73. doi: 10.1007/s10902-025-00907-2

[10] Fusi G Palmiero M Lavolpe S Colautti L Crepaldi M Antonietti A . Aging and psychological well-being: the possible role of inhibition skills. Healthcare. (2022) 10:1477. doi: 10.3390/healthcare10081477 36011134 PMC9408411

[11] Lee YJ Pang SF Choi HC Yu M Lee H. Exploring the mechanisms of tourist well-being: an application of cognitive appraisal theory and self-determination theory. Tour Hosp Res. (2026) 26:359–72. doi: 10.1177/14673584241293900

[12] Rozanski A. The pursuit of health: a vitality based perspective. Prog Cardiovasc Dis. (2023) 77:14–24. doi: 10.1016/j.pcad.2023.04.001 37037404

[13] Handlovsky I Wonsiak T Amato AT. Older, self-identifying gay men's conceptualisations of psychological well-being (PWB): a Canadian perspective. Nursing Philosophy. (2024) 25:e12466. doi: 10.1111/nup.12466 37828850

[14] Waddell C Van Doorn G Power G Statham D. From successful ageing to ageing well: a narrative review. Gerontologist. (2025) 65:gnae109. doi: 10.1093/geront/gnae109 39139052 PMC11638766

[15] Chang L Moyle BD Filep S Dupre K Zhang Y . Navigating life-course transitions through tourism: a dual pathway framework of senior wellbeing and resilience. J Travel Res. (2025) 00472875251384289. doi: 10.1177/00472875251384289

[16] Hou H Liu I Kong F Ni S. Computational positive psychology: advancing the science of wellbeing in the digital era. J Posit Psychol. (2025) 20:1–14. doi: 10.1080/17439760.2024.2362443

[17] Li X Li K Liu X Wu Y Huo B Wang Y . culturally integrative occupational therapy practice model based on traditional Chinese health culture in Mainland China. Hong Kong J Occup Ther. (2025) 38:155–66. doi: 10.1177/15691861251364399 40814333 PMC12343536

[18] Boaventura P Jaconiano S Ribeiro F. Yoga and Qigong for health: two sides of the same coin? Behav Sci. (2022) 12:222. doi: 10.3390/bs12070222 35877292 PMC9312231

[19] Fei LL Zhao SX Chen YF Hao CF Xin YJ. Exercise and sleep health in college students: efficacy, mechanisms, and implications for practice. World J Psychiatry. (2025) 15:108884. doi: 10.5498/wjp.v15.i10.108884 41112608 PMC12531955

[20] Chang CI Yeh ML Liao J. Chan-Chuang Qigong with breathing meditation improves quality of life and interoceptive awareness in patients with breast cancer: a randomised controlled trial. Support Care Cancer. (2023) 31:140. doi: 10.1007/s00520-023-07578-w 36707489

[21] Tsai YN Chang YH Su YC Lee SM Hsiao CH Lin CK . A community-based Daoyin program for health promotion: effects of the Qi and mind harmonizing method on body constitution for the health of older adults. Front Public Health. (2026) 13:1644273. doi: 10.3389/fpubh.2025.1644273 41561842 PMC12812638

[22] Xie F Guan C Gu Y You Y Yao F. Effects of the prolong life with nine turn method (Yan Nian Jiu Zhuan) Qigong on brain functional changes in patients with chronic fatigue syndrome in terms of fatigue and quality of life. Front Neurol. (2022) 13:866424. doi: 10.3389/fneur.2022.866424 35911899 PMC9326262

[23] Liu D Zhou B Wen Z Zhang Y. From healing and martial roots to global health practice: reimagining Tai Chi (Taijiquan) in the modern public fitness movement. Front Public Health. (2025) 13:1677470. doi: 10.3389/fpubh.2025.1677470 40963652 PMC12436410

[24] Mind the Motion: Feasibility and Effects of a Qigong Intervention on Interoception and Well-Being in Young Adults. https://www.mdpi.com/2227-9032/14/2/202 doi: 10.3390/healthcare14020202 41595337 PMC12841306

[25] Mendo B Gonçalves M Lopes L Matos LC Machado J. Can yoga, Qigong, and Tai Chi breathing work support the psycho-immune homeostasis during and after the covid-19 pandemic? a narrative review Healthcare. (2022) 10:1934. doi: 10.3390/healthcare10101934 36292380 PMC9601556

[26] Geisz S. Qigong, philosophical reading, and the cultivation of attention: Chinese contemplative body practices and slow philosophy. Sport, Ethics and Philosophy. (2022) 16:165–79. doi: 10.1080/17511321.2020.1748098

[27] Ye W Li S. Digital protection analysis of national traditional sports health cultural heritage based on big data in the era of data cloud. Multimed Tools Appl. (2024) 83:69739–58. doi: 10.1007/s11042-024-18371-0

[28] Park M Song R Ju K Shin JC Seo J Fan X . Effects of Tai Chi and Qigong on cognitive and physical functions in older adults: systematic review, meta-analysis, and meta-regression of randomized clinical trials. BMC Geriatr. (2023) 23:352. doi: 10.1186/s12877-023-04070-2 37280512 PMC10242998

[29] Dong Y Pang D Xiang J Chao G Kuang X. Exploring the benefits of traditional Chinese exercises (Tai Chi and Qigong) on the anxiety and depression of older adults: a systematic review and meta-analysis. Medicine. (2025) 104:e41908. doi: 10.1097/MD.0000000000041908 40128068 PMC11936652

[30] Yeh GY Ahn A Clark J Irwin MR Kong J Lavretsky H . The Science of Tai Chi and Qigong as whole person health-part ii: evidence gaps and opportunities for future research and implementation. Journal of Integrative and Complementary Medicine. (2025) 31:521–34. doi: 10.1089/jicm.2024.0958 40229137 PMC12172645

[31] Qiu T Zhang G Zhou F Jiang H. The socialization of older adults with depression in group Qigong training, - Taijiquan, Baduanjin, Yi Jin Jing. Curr Psychol. (2024) 43:30417–28. doi: 10.1007/s12144-024-06636-8

[32] Martín-Rodríguez A Gostian-Ropotin LA Beltrán-Velasco AI Belando-Pedreño N Simón JA López-Mora C . Sporting mind: the interplay of physical activity and psychological health. Sports. (2024) 12:37. doi: 10.3390/sports12010037 38275986 PMC10819297

[33] Wei L Hu Y Tao Y Hu R Zhang L. The effects of physical exercise on the quality of life of healthy older adults in China: a systematic review. Front Psychol. (2022) 13:895373. doi: 10.3389/fpsyg.2022.895373 35800916 PMC9253880

[34] Sun W Li L Zhang Z Duan X Wei J Soh KL. Effects of traditional fitness qigong exercise on frailty status and overall well-being in frail or pre-frail patients: a systematic review and meta-analysis. Front Med. (2025) 12:1619729. doi: 10.3389/fmed.2025.1619729 40727533 PMC12301368

[35] Li W Hong S Chen Y Zhao Y Wang L. Factors influencing physical activity behavior in older adults with subjective cognitive decline: an empirical study using SEM and fsQCA methods. Front Public Health. (2024) 12:1409614. doi: 10.3389/fpubh.2024.1409614 39484360 PMC11526387

[36] Xiong X Zhang L Zhang E. The effects of Qigong exercise on sleep quality in older adults: a systematic review and meta-analysis. Front Public Health. (2025) 13:1664055. doi: 10.3389/fpubh.2025.1664055 41487630 PMC12757222

[37] Jin F Chen T. Community nursing on subjective well-being of the elderly: evidence from CLASS data. BMC Nurs. (2025) 24:757. doi: 10.1186/s12912-025-03205-7 40597969 PMC12211858

[38] Qiu X Liang J Luo L Huang X Yi G Xiong C . Patterns of social participation among older adults with chronic multimorbidity in the community: a qualitative study. Int J Nurs Stud. (2026) 174:105279. doi: 10.1016/j.ijnurstu.2025.105279 41260153

[39] Sun W. The cognitive effects and the underlying neurophysiological mechanisms of qigong in depressed older adults : a randomized controlled trial. (2025) https://theses.lib.polyu.edu.hk/handle/200/13793

[40] Zeng S Bailey RP Khosravi V Song Y Liu W Sweeney R. Studying Baduanjin Qigong: a bibliometric analysis of research trends and future prospects. International Sports Studies. (2025) 47:157–74. doi: 10.69665/iss.v47i2.98

[41] Morgan GS Willmott M Ben-Shlomo Y Haase AM Campbell RM A. life fulfilled: positively influencing physical activity in older adults – a systematic review and meta-ethnography. BMC Public Health. (2019) 19:362. doi: 10.1186/s12889-019-6624-5 30940111 PMC6444855

[42] Chen C Beaunoyer E Guitton MJ Wang J Chen C Beaunoyer E . Physical activity as a clinical tool against depression: opportunities and challenges. J Integr Neurosci. (2022) 21:132. doi: 10.31083/j.jin2105132 36137954

[43] Phoenix C Orr N. Pleasure: a forgotten dimension of physical activity in older age. Soc Sci Med. (2014) 115:94–102. doi: 10.1016/j.socscimed.2014.06.013 24955874

[44] Deci EL Eghrari H Patrick BC Leone DR. Facilitating internalization: the self-determination theory perspective. J Pers. (1994) 62:119–42. doi: 10.1111/j.1467-6494.1994.tb00797.x 8169757

[45] Zhang S Li J Zou J Ai Y Qin S Xiao X . Empowerment of the older adults in the context of Chinese culture: an evolutionary concept analysis. Front Psychol. (2023) 14:1271315. doi: 10.3389/fpsyg.2023.1271315 38022912 PMC10666161

[46] Roth LHO Bencker C Lorenz J Laireiter AR. Testing the validity of the broaden-and build theory of positive emotions: a network analytic approach. Front Psychol. (2024) 15:1405272. doi: 10.3389/fpsyg.2024.1405272 39380763 PMC11458511

[47] Shin K You S Sohn Y. The association between serious leisure and university students' life satisfaction: focusing on the broaden-and-build theory of positive emotions. Behavioral Sciences. (2025) 15:1677. doi: 10.3390/bs15121677 41464020 PMC12729602

[48] Seehagen S Bartnick C Kärtner J Krasko J Luhmann M Schaal N . Belong, broaden, and build: the role of positive emotions in early human development. Child Dev Perspect. (2025) 19:237–43. doi: 10.1111/cdep.70002

[49] Sun C Yang S Wang X Shao Y Huang X Qi H . Embodied cognition driven Qigong: a cross-sectional study and a pilot randomized controlled trial on managing depression and preventing relapse in substance dependence. Front Public Health. (2024) 12:1388887. doi: 10.3389/fpubh.2024.1388887 39555034 PMC11564176

[50] McLeod CJ. Using Integral Theory to Study the Effectiveness of Heart Math Biofeedback and Social-Emotional Learning in Adolescent Emotion Regulation. (2024) Available online at: https://hdl.handle.net/1880/120277 (Accessed September 10, 2026).

[51] Li W Liu T Sun C. Hunyuan zhuang improves interoception: evidence from an expert-novice study and a pilot randomized controlled trial. Sci Rep. (2026) 16:11872. doi: 10.1038/s41598-026-42253-0 41776288 PMC13066386

[52] Low SLK Ho GF Liu B Koh ES Fei Y Teo CS . Exploring Guolin Qigong (mind-body exercise) for improving cancer related fatigue in cancer survivors: a mixed method randomized controlled trial protocol. Integr Cancer Ther. (2024) 23:15347354241252698. doi: 10.1177/15347354241252698 38757745 PMC11102686

[53] Wan Z Liu X Yang H Li F Yu L Li L . Effects of health Qigong exercises on physical function on patients with parkinson's disease. J Multidiscip Healthc. (2021) 14:941–50. doi: 10.2147/JMDH.S303945 33953563 PMC8091455

[54] Gilchrist H Haynes A Oliveira JS Grunseit A Sherrington C Bauman A . The value of mind–body connection in physical activity for older people. J Aging Phys Act. (2022) 31:81–8. doi: 10.1123/japa.2021-0503 35894992

[55] Yiming Y Ma R Saiyidu Y. The mediating effect of psychological well-being on self-efficacy and career development of physical education major students. BMC Psychol. (2025) 13:862. doi: 10.1186/s40359-025-03168-z 40760499 PMC12323181

[56] Nawa NE Yamagishi N. Distinct associations between gratitude, self-esteem, and optimism with subjective and psychological well-being among Japanese individuals. BMC Psychol. (2024) 12:130. doi: 10.1186/s40359-024-01606-y 38454459 PMC10918921

[57] Engelen L Rahmann M de Jong E. Design for healthy ageing – the relationship between design, well-being, and quality of life: a review. Building Research & Information. (2022) 50:19–35. doi: 10.1080/09613218.2021.1984867

[58] Gong K Wang C Yin J. Effects of the natural environment on the subjective and psychological well-being of older people in the community in China. Buildings. (2024) 14:2854. doi: 10.3390/buildings14092854

[59] Wong MYC Ou K Chung PK Chui KYK Zhang C. The relationship between physical activity, physical health, and mental health among older Chinese adults: a scoping review. Front Public Health. (2023) 10:914548. doi: 10.3389/fpubh.2022.914548 36684983 PMC9853435

[60] Yang Q Zhang Y Li S. The impact of mind–body exercise on the quality of life in older adults: the chain mediation effect of perceived social support and psychological resilience. Front Public Health. (2024) 12:1446295. doi: 10.3389/fpubh.2024.1446295 39421821 PMC11484243

[61] Bonanno GA Chen S Galatzer-Levy IR. Resilience to potential trauma and adversity through regulatory flexibility. Nat Rev Psychol. (2023) 2:663–75. doi: 10.1038/s44159-023-00233-5

[62] Noto S. Perspectives on aging and quality of life. Healthcare. (2023) 11:2131. doi: 10.3390/healthcare11152131 37570372 PMC10418952

[63] Gianfredi V Nucci D Pennisi F Maggi S Veronese N Soysal P. Aging, longevity, and healthy aging: the public health approach. Aging Clin Exp Res. (2025) 37:125. doi: 10.1007/s40520-025-03021-8 40244306 PMC12006278

[64] Sars D. PE augmented mindfulness: a neurocognitive framework for research and future healthcare. Front Hum Neurosci. (2022) 16:899988. doi: 10.3389/fnhum.2022.899988 36082227 PMC9446465

[65] Nicholson WC Sapp M Karas EM Duva IM Grabbe L. The body can balance the score: using a somatic self-care intervention to support well-being and promote healing. Healthcare. (2025) 13:1258. doi: 10.3390/healthcare13111258 40508871 PMC12154529

[66] Larkey L Kim W James D Kishida M Vizcaino M Huberty J . Mind-body and psychosocial interventions may similarly affect heart rate variability patterns in cancer recovery: implications for a mechanism of symptom improvement. Integr Cancer Ther. (2020) 19:1534735420949677. doi: 10.1177/1534735420949677 32783546 PMC7425257

[67] Lee YH Chang YP Lee JT Lee DC Huang EY Lai LJT. Heart rate variability as an indicator of the beneficial effects of Qigong and mindfulness training on the mind–body well-being of cancer survivors. Support Care Cancer. (2022) 31:59. doi: 10.1007/s00520-022-07476-7 36534354 PMC9761690

[68] Leung KCW Yang YJ Hui SSC Woo J. Mind-body health benefits of traditional Chinese qigong on women: a systematic review of randomized controlled trials. Evidence-Based Complementary and Alternative Medicine. (2021) 2021:7443498. doi: 10.1155/2021/7443498 34567220 PMC8457943

[69] Dong S Ge H Su W Guan W Li X Liu Y . Enhancing psychological well-being in college students: the mediating role of perceived social support and resilience in coping styles. BMC Psychol. (2024) 12:393. doi: 10.1186/s40359-024-01902-7 39010140 PMC11250932

[70] Peng B Chen W Wang H Yu T. How does physical exercise influence self-efficacy in adolescents? A study based on the mediating role of psychological resilience. BMC Psychol. (2025) 13:285. doi: 10.1186/s40359-025-02529-y 40119462 PMC11927186

[71] Bak W Dutkiewicz D Brudek P. Relationship between self-assessed health and life satisfaction in older adults: the moderating role of ego-resiliency. Ageing & Society. (2023) 43:2335–46. doi: 10.1017/S0144686X21001689

[72] Zheng Y Lin Y Zheng Q. Traditional physical exercise and depression in older adults: the mediating role of interoceptive awareness and the moderating effect of perceived stress. Front Public Health. 14:1766902. doi: 10.3389/fpubh.2026.1766902 41869607 PMC12999434

[73] Acoba EF. Social support and mental health: the mediating role of perceived stress. Front Psychol. (2024) 15:1330720. doi: 10.3389/fpsyg.2024.1330720 38449744 PMC10915202

[74] Bi X He H Lin H Fan X. Influence of social support network and perceived social support on the subjective wellbeing of mothers of children with autism spectrum disorder. Front Psychol. (2022) 13:835110. doi: 10.3389/fpsyg.2022.835110 35401352 PMC8989138

[75] Nyqvist F Forsman AK Giuntoli G Cattan M. Social capital as a resource for mental well-being in older people: a systematic review. Aging Ment Health. (2013) 17:394–410. doi: 10.1080/13607863.2012.742490 23186534

[76] Xu T Mao Y Wang Y Gong Y Yu E. Recent advances in multimodal non-pharmacological interventions for late-life depression. Neuropsychiatr Dis Treat. (2025) 21:2525–44. doi: 10.2147/NDT.S558969 41292672 PMC12642798

[77] Lin CY Wei TT Wang CC Chen WC Wang YM Tsai SY. Acute physiological and psychological effects of qigong exercise in older practitioners. Evid Based Complement Altern Med. (2018) 2018:4960978. doi: 10.1155/2018/4960978 29805467 PMC5902057

[78] Wong MYC Zhang CQ Zhao Y Hu C Ou K. Effectiveness of resistance training on resilience in Hong Kong Chinese older adults. Cogent Psychology. (2024) 11:2426873. doi: 10.1080/23311908.2024.2426873

[79] Fan F Gou H Li P Xiang L Du J. The relationship between Tai Chi practice, psychological resilience, and depression in older adults: an integrated study of mediation and network analysis. Front Public Health. (2026) 14:1777392. doi: 10.3389/fpubh.2026.1777392 41859273 PMC12995767

[80] Xiang L Gou H Sun Q Fan F Hu C. Adverse childhood experiences and cyber aggression among college students: the mediating role of psychological resilience and the moderating role of physical exercise. Front Psychiatry. (2026). doi: 10.3389/fpsyt.2026.1943434 PMC13506935 42656723

[81] Oh YT Ryu MA Uhm JP. The role of intramural combat martial arts in enhancing well-being among international students: a combined theoretical approach. Front Psychiatry. (2025) 16:1582731. doi: 10.3389/fpsyt.2025.1582731 40291518 PMC12023476

[82] Gaviano L Pili R Petretto AD Berti R Carrogu GP Pinna M . Definitions of ageing according to the perspective of the psychology of ageing: a scoping review. Geriatrics. (2024) 9:107. doi: 10.3390/geriatrics9050107 39311232 PMC11417832

[83] Baltes MM Carstensen LL. The process of successful ageing. Ageing & Society. (1996) 16:397–422. doi: 10.1017/S0144686X00003603

[84] Walker A. A strategy for active ageing. Available online at: https://onlinelibrary.wiley.com/doi/10.1111/1468-246X.00118

[85] Beard JR Officer A Carvalho IA de Sadana R Pot AM Michel J-P . The World report on ageing and health: a policy framework for healthy ageing. Lancet. (2016) 387:2145–54. doi: 10.1016/S0140-6736(15)00516-4 26520231 PMC4848186

[86] Bar-Tur L. Fostering well-being in the elderly: translating theories on positive aging to practical approaches. Front Med. (2021) 8:517226. doi: 10.3389/fmed.2021.517226 33898472 PMC8062922

[87] Yin Z Martinez CE Li S Martinez M Peng K Land WM . Adapting Chinese qigong mind-body exercise for healthy aging in older community-dwelling low-income latino adults: pilot feasibility study. JMIR Aging. (2021) 4:e29188. doi: 10.2196/29188 34723824 PMC8593812

[88] Menassa M Stronks K Khatami F Díaz ZMR Espinola OP Gamba M . Concepts and definitions of healthy ageing: a systematic review and synthesis of theoretical models. eClinicalMedicine. (2023) 56: doi: 10.1016/j.eclinm.2022.101821 36684393 PMC9852292

[89] Li RH. Reliability and validity of a shorter chinese version for ryff's psychological well-being scale. Health Educ J. (2014) 73:446–52. doi: 10.1177/0017896913485743

[90] Connor KM Davidson JRT. Development of a new resilience scale: the Connor-Davidson resilience scale (CD-RISC). Depress Anxiety. (2003) 18:76–82. doi: 10.1002/da.10113 12964174

[91] Yu XN Zhang JX A. comparison between the ego-resiliency scale and the Connor-Davidson resilience scale. Psychol Sci. (2007) 30:1169–71.

[92] Blumenthal JA Zhu Y Koch GG Smith PJ Watkins LL Hinderliter AL . The modifying effects of social support on psychological outcomes in patients with heart failure. Health Psychology. (2019) 38:502–8. doi: 10.1037/hea0000716 30998063 PMC6599535

[93] Zhou C Wu D Li Y Yang HH Man S Chen M. The role of student engagement in promoting teachers' continuous learning of TPACK: based on a stimulus-organism-response framework and an integrative model of behavior prediction. Educ Inf Technol. (2023) 28:2207–27. doi: 10.1007/s10639-022-11237-8

[94] Yoon SI Park HY Park C Pyun J Yoo JH Song GJ . Effectiveness of mindfulness and qigong training for self-healing in patients with Hwabyung and depressive disorder: a randomized controlled trial. Front Psychiatry. (2025) 16:1508937. doi: 10.3389/fpsyt.2025.1508937 40585550 PMC12202437

[95] Lin J Gao Y. Fang, Guo Y, Li M, Zhu Y, You R, et al. Effects of qigong exercise on the physical and mental health of college students: a systematic review and meta-analysis. BMC Complement Med Ther. (2022) 22:287. doi: 10.1186/s12906-022-03760-5 36348349 PMC9641907

[96] Yang H Li B Feng L Zhang Z Liu X. Effects of health qigong exercise on upper extremity muscle activity, balance function, and quality of life in stroke patients. Front Neurosci. (2023) 17:1208554. doi: 10.3389/fnins.2023.1208554 37539378 PMC10394517

[97] Daniels K Lemmens R Knippenberg E Marinus N Vonck S Baerts J . Promoting physical activity and a healthy active lifestyle in community-dwelling older adults: a design thinking approach for the development of a mobile health application. Front Public Health. (2023) 11:1280941. doi: 10.3389/fpubh.2023.1280941 38106904 PMC10724027

[98] Tsai TH Wong AM Lee HF Tseng KC A. study on the motivation of older adults to participate in exercise or physical fitness activities. Sustainability. (2022) 14:6355. doi: 10.3390/su14106355

[99] Sun J Zhuo J Chu H Wang J Chen T Li B . Effects of 3-month Qigong exercise on heart rate variability and respiration in anxious college students. Scand J Med Sci Sports. (2024) 34:e14521. doi: 10.1111/sms.14521 37815004

[100] Qi W Yu H Wilczyńska D A. cross-sectional study on the psychological effects of different exercises on elderly women. Brain Sci. (2025) 15:918. doi: 10.3390/brainsci15090918 41008279 PMC12468438

[101] Dong Y Zhang X Zhao R Cao L Kuang X Yao J. The effects of mind-body exercise on anxiety and depression in older adults: a systematic review and network meta-analysis. Front Psychiatry. (2024) 15:1305295. doi: 10.3389/fpsyt.2024.1305295 38384592 PMC10879425

[102] Zhang B Huang Y. Practicing Taichi together in the park: a case study of Taichiscape and older people's wellbeing. Popul Space Place. (2023) 29:e2696. doi: 10.1002/psp.2696

[103] van Dam K. Individual stress prevention through Qigong. Int J Environ Res Public Health. (2020) 17:7342. doi: 10.3390/ijerph17197342 33050017 PMC7579037

[104] DiGirolamo MA Kibrislioglu Uysal N McCall EC Isaacowitz DM. Attention-focused emotion regulation in everyday life in adulthood and old age. Emotion. (2023) 23:633–50. doi: 10.1037/emo0001158 35951383

[105] Huang X Wang X Shao Y Lin A Zhang Z Qi H . Effects of health qigong exercise on sleep and life quality in patients with drug abuse. Hong Kong Journal of Occupational Therapy. (2023) 36:13–9. doi: 10.1177/15691861231156002 37332297 PMC10273793

[106] Watkins P Hill AM Thaver IK Burton E. Staying strong together: older people engaging in resistance training with a peer. J Aging Phys Act. (2021) 29:807–13. doi: 10.1123/japa.2020-0211 33714192

[107] Jiang Z Ren H Sun Q Zhang H Zhou S. Attitude towards aging and anxiety in the relationship between social alienation and depression in Chinese rural empty-nest older adults: a chained mediation modeling analysis. BMC Public Health. (2025) 25:1435. doi: 10.1186/s12889-025-22712-5 40241111 PMC12001739

[108] Wang X Shao S Cheng H Blain SD Tan Y Jia L. Effects of cognitive flexibility on dynamics of emotion regulation and negative affect in daily life. Anxiety, Stress, & Coping. (2025) 38:365–78. doi: 10.1080/10615806.2024.2423154 39505488

[109] van Dierendonck D Lam H. Interventions to enhance eudaemonic psychological well-being: a meta-analytic review with Ryff's scales of psychological well-being. Appl Psychol Health Well-Being. (2023) 15:594–610. doi: 10.1111/aphw.12398 36073601

[110] Bastos A Monteiro J Barbosa R Pimentel H Silva S Faria C. Engagement with life and psychological well-being in late adulthood: Findings from community-based programs in Portugal. PLoS ONE. (2023) 18:e0286115. doi: 10.1371/journal.pone.0286115 37205691 PMC10198493

[111] Chang PS Lu Y Nguyen CM Suh Y Luciani M Ofner S . Effects of qigong exercise on physical and psychological health among African Americans. West J Nurs Res. (2021) 43:551–62. doi: 10.1177/0193945920959067 32942949 PMC8159432

[112] Antonelli M Donelli D. Evaluating qigong as integrative support for COVID-19 and Long-COVID-19 rehabilitation: a systematic review. Front Psychol. (2024) 15:1403130. doi: 10.3389/fpsyg.2024.1403130 38807958 PMC11130475

[113] Liu J Zhao J Wang Z. The effect of peer support on psychological rehabilitation in injured collegiate athletes: the mediating roles of resilience and perceived stress. Front Psychol. (2025) 16:1567812. doi: 10.3389/fpsyg.2025.1567812 41112547 PMC12528014

[114] Gupta S McCarthy PJ. The sporting resilience model: a systematic review of resilience in sport performers. Front Psychol. (2022) 13:1003053. doi: 10.3389/fpsyg.2022.1003053 36619099 PMC9811683

[115] Zhu Z Hu X Zhang B. The role of resilience in navigating work stress and achieving daily work goals. J Organ Behav. (2025) 46:1107–19. doi: 10.1002/job.2839

[116] Schneider S von Winning A Grüger F Anderer S Hoffner R Anderson L. Physical activity, climate change and health—a conceptual model for planning public health action at the organizational level. Int J Environ Res Public Health. (2022) 19:4664. doi: 10.3390/ijerph19084664 35457530 PMC9025182

[117] Zapater-Fajarí M Crespo-Sanmiguel I Pulopulos MM Hidalgo V Salvador A. Resilience and psychobiological response to stress in older people: the mediating role of coping strategies. Front Aging Neurosci. (2021) 13:632141. doi: 10.3389/fnagi.2021.632141 33692681 PMC7937969

[118] Çavdar S Kayhan Koçak FO Piotrowicz K Okyar Baş A Zhu L Salis F . Factors associated with self-perceptions of ageing among geriatric healthcare professionals: a study by the early career geriatricians initiative group of EuGMS. Eur Geriatr Med. (2026) doi: 10.1007/s41999-026-01421-1 41663873

[119] Yun A Cai Y Hu Y. The relationship between Chinese vocational college students' adverse childhood experience and meaning in life: the mediating role of self-esteem and the moderating role of humorous coping. Front Psychol. (2025) 16:1467780. doi: 10.3389/fpsyg.2025.1467780 40309205 PMC12040953

[120] Jiang Y Li P Yang Y Liu L Liu H Zhu Y . Neural mechanisms and social support for mental health in old age by martial arts exercise. Front Psychol. (2026) 16:1733310. doi: 10.3389/fpsyg.2025.1733310 41583770 PMC12827584

[121] Zhang G Cao Y Yan Z. Editorial: mindfulness, mind-body exercises, and health promotion. Front Psychol. (2025) 16:1559535. doi: 10.3389/fpsyg.2025.1559535 39968196 PMC11832519

[122] He J Wang H. The impact of physical exercise on subjective well-being in migrant elderly: the chain mediation of social support and psychological capital. Curr Psychol. (2025) 44:16771–82. doi: 10.1007/s12144-025-08390-x

[123] Whitaker CB. Qigong as Holistic Healing to Counter Health Harms of Social Injustice. Holistic Education Review. (2025) 5: Available online at: https://her.journals.publicknowledgeproject.org/index.php/her/article/view/3320 (Accessed April 27, 2026).

[124] Singh S Nguyen T Deleemans J Oberoi D Piedalue KA Carlson LE . phenomenological qualitative exploration of mind-body therapy use and effectiveness among young, middle, and older adult cancer survivors. Integr Cancer Ther. (2024) 23:15347354241253847. doi: 10.1177/15347354241253847 38767143 PMC11107309

[125] Liu X Li R Cui J Liu F Smith L Chen X . The effects of tai chi and qigong exercise on psychological status in adolescents: a systematic review and meta-analysis. Front Psychol. (2021) 12:746975. doi: 10.3389/fpsyg.2021.746975 34899487 PMC8652254

[126] Susan JL. The utilization of Qigong as a non-pharmacological group activity and its impact on mood and well-being. Rutgers University - School of Nursing - RBHS. (2021).

[127] Bias in cross-sectional analyses of longitudinal mediation. Available online at: https://psycnet.apa.org/record/2007-03329-002 (Accessed July 26, 2026).

[128] Cao L Dong X Qi K Zhou C Chen A. Efficacy of Taiji Stick exercise on sleep quality and anxiety in older adults: a randomized controlled trial. Front Psychol. 17:1760046. doi: 10.3389/fpsyg.2026.1760046 41798036 PMC12963296

[129] Han S Li B Wang G Ke Y Meng S Li Y . Physical fitness, exercise behaviors, and sense of self-efficacy among college students: a descriptive correlational study. Front Psychol. (2022) 13:932014. doi: 10.3389/fpsyg.2022.932014 35910985 PMC9335150

[130] Ye Y Wan M Lin H Xia R He J Qiu P . Effects of Baduanjin exercise on cognitive frailty, oxidative stress, and chronic inflammation in older adults with cognitive frailty: a randomized controlled trial. Front Public Health. (2024) 12:1385542. doi: 10.3389/fpubh.2024.1385542 38846613 PMC11153822

