# Supplementary material for: Health Qigong engagement scale and wellbeing in Chinese older adults
Source: Front Public Health. 2026 Sep 23;14:1889948. doi: 10.3389/fpubh.2026.1889948 (PMC13642148; doi:10.3389/fpubh.2026.1889948)
Supplement: Supplementary file 1 [file Data_Sheet_1.docx]

Supplementary Material

# 1 Supplementary Figures and Tables

## 1.1 Supplementary Tables

Item analysis: We performed critical-ratio (CR) analysis and item–total correlation analysis, and removed items with CR < 3.0 or item–total correlation < 0.40. The results are presented in Table 1.

**Table 1.** Item analysis.

| Dimension | items | t | r |
| --- | --- | --- | --- |
| Continuous Behavioral Engagement | I regularly participate in Health Qigong practice. | 21.779*** | 0.689*** |
|  | Over the past period of time, I have basically been able to maintain the habit of practicing Health Qigong. | 25.652*** | 0.722*** |
|  | Even when I have other things to do in daily life, I still try to arrange time to practice Health Qigong. | 23.872*** | 0.720*** |
|  | My participation in Health Qigong is not just a temporary impulse, but something I can persist in continuously. | 22.605*** | 0.707*** |
|  | Health Qigong has become a part of my daily life. | 20.453*** | 0.676*** |
|  | I maintain a certain frequency of Health Qigong practice every week. | 22.489*** | 0.688*** |
| Normative Focused Engagement | When practicing Health Qigong, I pay attention to whether my movements are performed in a standardized way. | 20.870*** | 0.694*** |
|  | During practice, I consciously coordinate my breathing rhythm. | 20.079*** | 0.709*** |
|  | When practicing Health Qigong, I am able to complete the whole set of movements with relatively focused attention. | 21.849*** | 0.689*** |
|  | I pay attention to the coordination among movements, breathing, and bodily sensations. | 21.476*** | 0.713*** |
|  | When I notice that my movements are not standard, I actively adjust them. | 20.459*** | 0.707*** |
|  | I am willing to seriously learn the key movement techniques and practice methods of Health Qigong. | 22.490*** | 0.698*** |
| Autonomous Persistent Engagement | Even without being reminded by others, I will take the initiative to practice Health Qigong. | 22.407*** | 0.683*** |
|  | I will proactively adjust my Health Qigong practice arrangements according to my physical condition. | 24.309*** | 0.719*** |
|  | When weather, venue, or other conditions are inconvenient, I still try to find ways to continue practicing. | 23.224*** | 0.687*** |
|  | Even if I temporarily interrupt my practice for a period of time, I will resume Health Qigong practice again. | 22.951*** | 0.693*** |
|  | I am willing to make time arrangements in order to persist in practicing Health Qigong. | 22.876*** | 0.685*** |
|  | I actively learn about Health Qigong practice content that is suitable for me. | 18.373*** | 0.668*** |

Note: **p*< 0.05, ***p* < 0.01, ****p*< 0.001. The same notation applies below.

Internal consistency: The overall Cronbach’s a for the scale was 0.832. Cronbach’s a for each dimension are shown in Table 2.

**Table 2.** Summary of reliability analysis.

|  | number of terms | Cronbach’s a |
| --- | --- | --- |
| Continuous Behavioral Engagement | 6 | 0.956 |
| Normative Focused Engagement | 6 | 0.956 |
| Autonomous Persistent Engagement | 6 | 0.959 |
| Overall | 18 | 0.938 |

Exploratory Factor Analysis (EFA): Principal component analysis with varimax rotation was conducted. The KMO value exceeded 0.70, and Bartlett’s test of sphericity was significant (*p*< 0.001). Three common factors with eigenvalues greater than 1 were extracted, accounting for a cumulative variance of over 60%. All items had factor loadings above 0.55 with no cross-loadings. The results are shown in Table 4.

**Table 3.** KMO and Bartlett’s Test

| Kaiser-Meyer-Olkin Measure of Sampling Adequacy. |  | 0.938 |
| --- | --- | --- |
| Bartlett's Test of Sphericity | Approx. Chi-Square | 6462.500 |
|  | df | 153 |
|  | Sig. | 0.000 |

**Table 4.** Rotated component matrix from exploratory factor analysis

| Item | Factor | | |
| --- | --- | --- | --- |
|  | 1 | 2 | 3 |
| Q1 | 0.892 |  |  |
| Q2 | 0.890 |  |  |
| Q3 | 0.895 |  |  |
| Q4 | 0.901 |  |  |
| Q5 | 0.885 |  |  |
| Q6 | 0.908 |  |  |
| Q7 |  |  | 0.908 |
| Q8 |  |  | 0.847 |
| Q9 |  |  | 0.893 |
| Q10 |  |  | 0.862 |
| Q11 |  |  | 0.858 |
| Q12 |  |  | 0.854 |
| Q13 |  | 0.883 |  |
| Q14 |  | 0.894 |  |
| Q15 |  | 0.886 |  |
| Q16 |  | 0.892 |  |
| Q17 |  | 0.879 |  |
| Q18 |  | 0.868 |  |

Extraction Method: Principal Component Analysis.

Rotation Method: Varimax with Kaiser Normalization.

a Rotation converged in 5 iterations.

## 1.2 Supplementary Figures

Confirmatory Factor Analysis (CFA): A three-factor model was constructed using AMOS (Figure 1). The fit indices ($\chi$^2^/df = 1.965, RMSEA = 0.055, SRMR = 0.052, CFI = 0.980, TLI = 0.977) indicated good model fit and reliable structural validity.

**Figure 1.** Confirmatory factor analysis model.
